# Accuracy of the Tibial Component Alignment by Extramedullary System Using Simple Radiographic References in Total Knee Arthroplasty

**DOI:** 10.3390/medicina58091212

**Published:** 2022-09-02

**Authors:** Jin-Ho Cho, Jun Young Choi, Sung-Sahn Lee

**Affiliations:** Department of Orthopedic Surgery, Ilsan Paik Hospital, Inje University School of Medicine, Goyang-si 10380, Korea

**Keywords:** total knee arthroplasty, tibial component alignment, radiographic references, extramedullary system

## Abstract

*Background and Objectives*: The tibial component alignment is an important issue for the longevity of total knee arthroplasty (TKA). The purpose of our study was to investigate the usefulness of proximal tibial references determined by pre-operative radiography and intraoperative C-arm-guided hip and ankle center marking for the extramedullary guided tibial cut in mild (<10°) and severe (≥10°) varus knee TKA. *Materials and Methods*: A total of 150 consecutive patients (220 cases) who underwent total knee arthroplasty who were recruited from July 2011 to April 2017 were reviewed retrospectively. Before surgery, the proximal tibial reference point and medio-lateral cut thickness difference were identified. Then, hip and ankle centers were checked using a C-arm intensifier intraoperatively. The hip–knee–ankle (HKA) alignment and medial proximal tibial angle (MPTA) were assessed pre-operatively and post-operatively. More than 3° varus or valgus of HKA alignment or tibial component angle was defined as an outlier. *Results*: Mean follow-up duration was 26.9 months. Among 220 cases, 111 cases are classified as mild varus group and 109 cases are classified as severe varus group. The HKA alignment is significantly improved (*p* < 0.001). The average tibial component angle after surgery is 90.1°. A total of 21 cases (9.5%) and 3 cases (1.4%) are classified as outliers of HKA alignment and MPTA, respectively. Among MPTA outliers, one case is in the mild varus group and two cases are in the in severe varus group (*p* = 0.62). *Conclusion*: Measurement of proximal tibial radiographic references and checking the C-arm-guided intraoperative hip and ankle center could be helpful to obtain the favorable coronal position of the tibial component in the extramedullary guided tibial cut.

## 1. Introduction

Kinematic alignment was recently investigated as an alternative to mechanically aligned total knee arthroplasty (TKA) [1]. Several studies demonstrate that kinematically aligned TKA shows similar or better clinical outcomes due to less disruption of the native soft tissue envelope [2,3]. As a similar theoretical background, some studies show that slight under-corrected TKA results in better clinical outcomes than neutrally aligned TKA [4]. However, many studies suggest that post-operative coronal alignment is associated with implant survival; in particular, tibial component varus position is strongly correlated to implant loosening [5,6,7].

**Figure 1:**
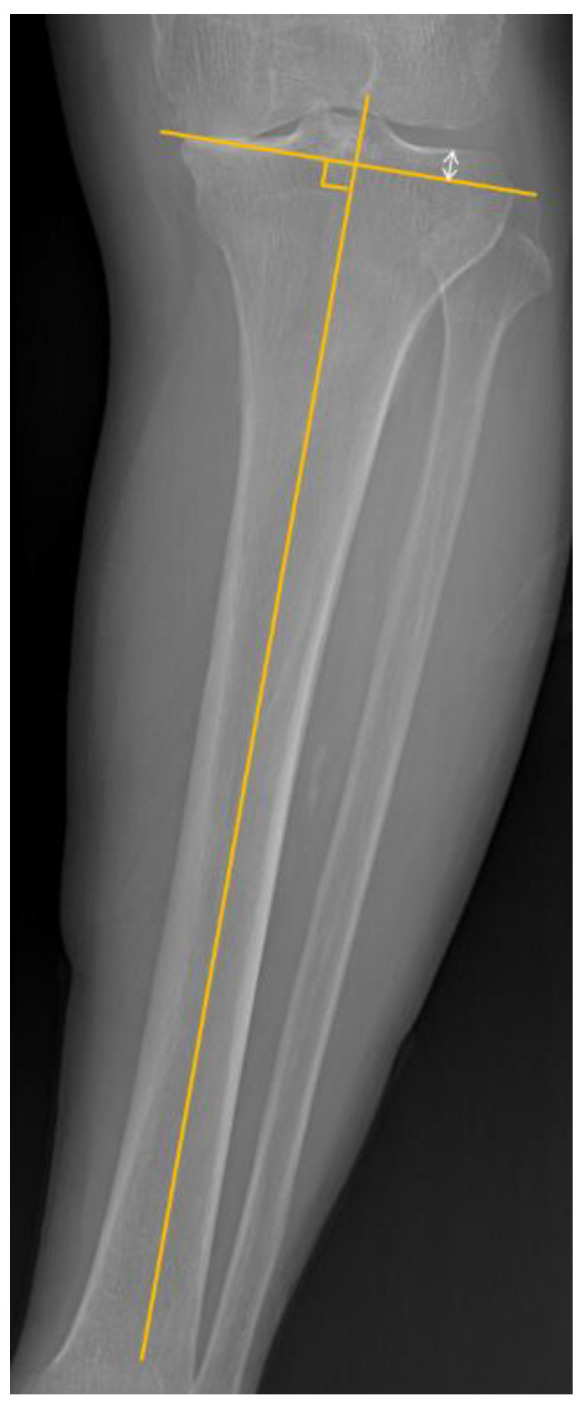
Proximal tibial medio-lateral (ML) cut thickness difference. The lines were drawn (1) along the anatomical axis of the tibia and (2) perpendicular to first line (starting from medial condylar edge). The *white arrow line* indicates proximal tibial ‘ML cut thickness difference’.

To enhance post-operative alignment, the computer navigation instruments or patient-specific implants (PSI) can be used [8,9,10]. However, these devices are not always available and have additional costs, therefore, many surgeons perform conventional jig-based TKA—intramedullary guided distal femoral cut and extramedullary guided proximal tibia cut. Various anatomical landmarks (extensor hallucis longus, dorsal pedis artery, intermalleolar point, anterior tibial border, intercondylar eminence) are used as a reference for extramedullary alignment to enhance tibial component coronal alignment [11,12,13]. Thippana et al. [14] reported that using the line that connects the proximal tibial reference point defined by pre-operative radiography and ankle center was helpful to set the extramedullary guide. In addition to this landmark, we thought pre-determined proximal tibial medio-lateral (ML) cut thickness difference by radiograph (Figure 1) and the intraoperative C-arm-intensifier-guided hip and ankle center marking method are helpful to enhance tibial component position.

The purpose of this study was (1) to investigate the usefulness of proximal tibial references determined by pre-operative radiography and intraoperative C-arm-guided hip and ankle center marking, and (2) to compare radiographic measurements between the patients with pre-operative mild varus (<10°) and severe varus (≥10°) deformity. It was hypothesized that favorable coronal alignment of the tibial component might be shown by this method, regardless of the severity of pre-operative varus deformity.

## 2. Materials and Methods

### 2.1. Patients

This study is a retrospectively designed study. From July 2011 to April 2017, the patients who underwent primary TKA surgery with the same method for degenerative osteoarthritis with varus deformity were reviewed. The exclusion criteria were as follows: patients who (1) followed up for less than 1 year, (2) had undergone other previous bony procedure, such as osteotomy, and (3) diagnosed systemic arthritis such as rheumatoid arthritis. The enrolled patients were divided into mild varus group (<10°) and severe varus group (≥10°). based on pre-operative hip–knee–ankle (HKA) alignment as the definition of previous studies [15,16]. Written informed consents were obtained from all patients before enrolling them in the study. This study was approved by the IRB of the authors’ affiliated institutions (ISPAIK 2019-02-014 at 20 February 2019).

### 2.2. Radiographic and Clinical Assessment

**Figure 2:**
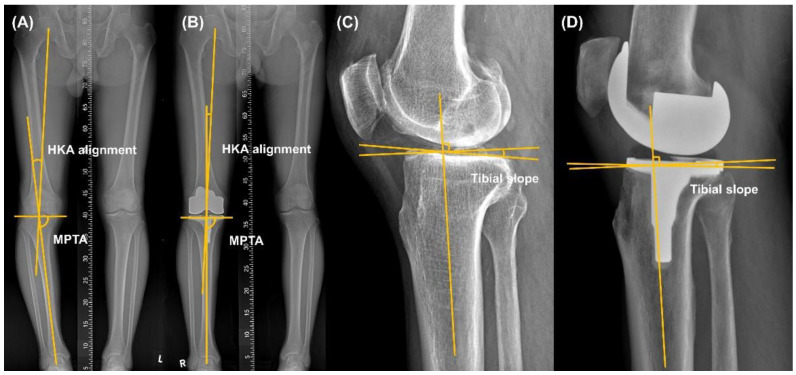
Measurement of the pre- and post-operative hip–knee–ankle (HKA) alignment, medial proximal tibial angle (MPTA, (**A**,**B**)) and tibial slope (**C**,**D**).

We analyzed the HKA alignment taken from the pre-operative and 1 year follow-up low extremity whole radiography [17,18]. As in previous studies, more than 3° varus or valgus of HKA alignment was defined as an outlier [4,19]. The medial proximal tibial angle (MPTA) is the medial angle of intersection between the anatomical axis of the tibia and the horizontal axis of the proximal tibia (pre-operative measurement) or tibial component (post-operative measurement). Refs. [20,21] are same as HKA alignment, more than 3° varus or valgus of tibial component angle was defined as an outlier [4,19]. The tibial slope was also measured as previously described [22,23] (Figure 2).

All radiographs were measured using Marosis software (INFINITT Healthcare, Seoul, Korea). The radiographs were evaluated by two independent orthopedic surgeons specializing in knee arthroplasty, who did not participate in the current study, to verify inter-observer reliability. The intra-observer reliability was checked by having the observers repeat the same measurements 6 weeks later. Intraclass correlation coefficients (ICCs) were used for intra-observer and inter-observer reliabilities.

The clinical parameters of pre-operation and last follow-up were evaluated by the following clinical scores: Hospital for Special Surgery (HSS) score [24] and Western Ontario Mac-Master University (WOMAC) Index [25,26]. In addition, the operative time and incision size were analyzed.

### 2.3. Pre-Operative Planning

**Figure 3:**
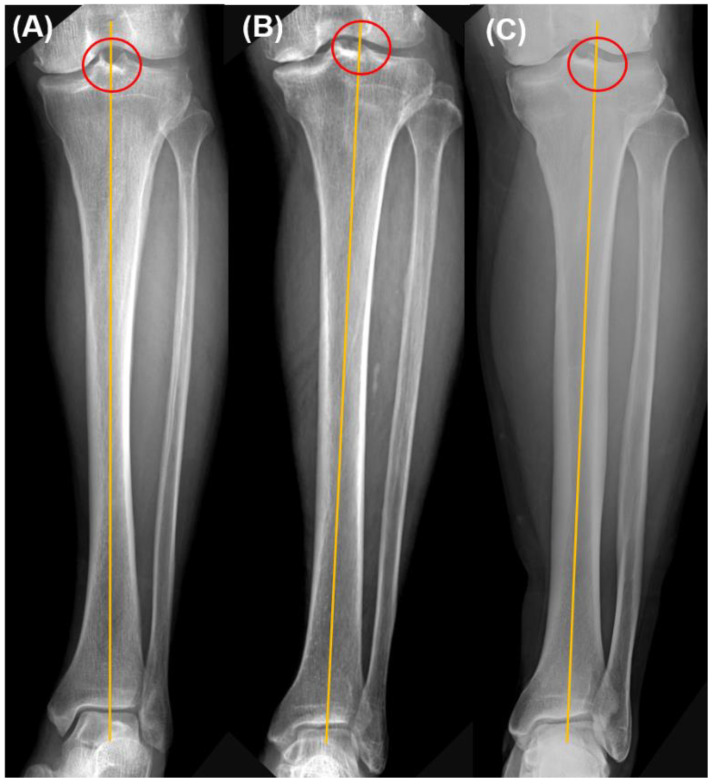
Proximal tibial reference point. These points were classified as 3 zones—(**A**) the center of intercondylar eminence, (**B**) in-between, and (**C**) lateral tibial spine.

Pre-operative tibial anteroposterior (AP) view was used to verify the proximal tibial reference point. The proximal tibial reference point is determined as the meeting point between the tibial anatomical axis and the proximal tibial joint line. Of the enrolled cases, all proximal tibial reference points existed between the center of intercondylar eminence and the lateral tibial spine. These points were classified as 3 zones—the center of intercondylar eminence, lateral tibial spine, and in-between (Figure 3).

The perpendicular line to the tibial anatomical axis was drawn from the edge of medial tibial condyle. The ML cut thickness difference of proximal tibia was measured (Figure 1).

### 2.4. Intraoperative Planning

**Figure 4:**
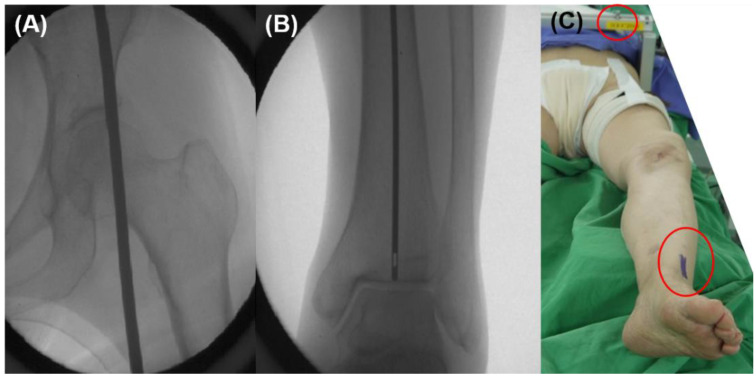
Marking hip and ankle centers. (**A**,**B**) Check hip and ankle centers using a metal rod under C-arm intensifier. (**C**) A hip center was marked using mobile peg in a pelvic stabilizer and an ankle center was marked with a marking pen.

After induction of anesthesia to the patients, the ankle center and hip center should be assessed under a C-arm intensifier to apply the pre-operative planning before draping. A long rod (High tibia osteotomy alignment rod, DePuy Synthes, Raynham, MA, USA) was used to confirm the hip center and ankle center. The patient was placed in a metal pelvic stabilizer with the mobile peg, so that the measured position did not change during surgery [27]. The hip center was marked with a mobile peg, and the end of the long rod was marked in the patient’s ankle using a marking pen (Figure 4).

### 2.5. Operative Procedure

All surgeries were performed by the senior author. In all cases, posterior, stabilized TKA implant was used (LEGION, Smith & Nephew, London, UK). Skin incision and medial parapatellar arthrotomy were performed. Both cruciate ligaments were removed from their femoral and tibial attachment sites and osteophytes were removed. Then, the intramedullary guided femoral cut was performed.

Extramedullary guided tibia cut was performed using pre- and intra-operatively checked references. The cartilages of lateral tibial condyle were removed. Extramedullary tibial cutting apparatus was installed using the proximal tibial reference point and marked ankle center. After that, we checked the ML thickness difference was similar between radiographic and intraoperative measurements using a cut thickness gauge.

**Figure 5:**
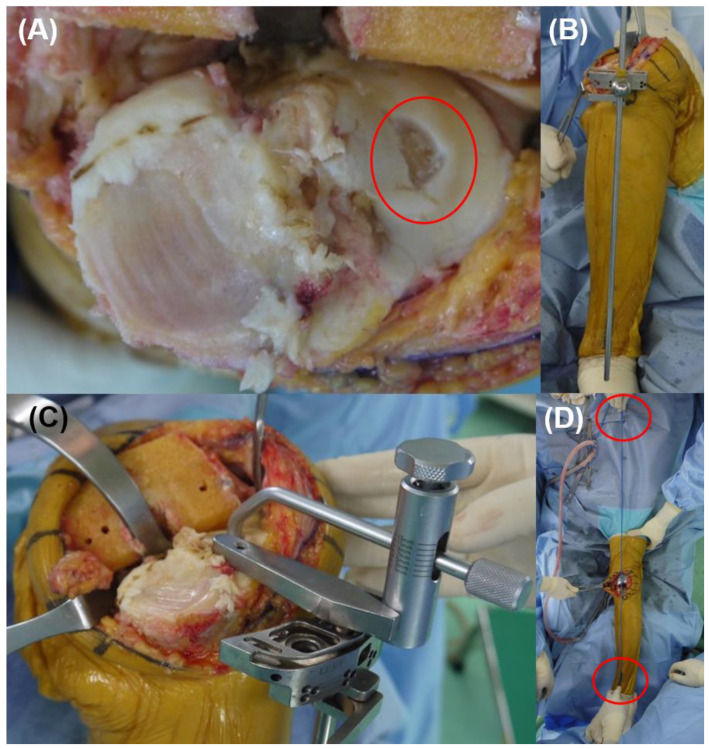
Operative procedure. (**A**) Gentle peeling of cartilage of the lateral condyle for precise intraoperative measurement. (**B**,**C**) Application of tibial cutting apparatus according to the ‘proximal tibial reference point’, ‘ankle center’, and pre-operatively measured medio-lateral cut thickness difference. (**D**) Check the limb and component coronal alignment using cable method.

After tibial cut and trial component insertion, marked ankle center and hip center should be connected by flexible cable (we used the cable-connecting electrocautery device) to check the tibial component coronal position and lower limb alignment. A proximal tibial cut was re-performed if the tibial component position was not perpendicular to cable (Figure 5).

After the surgery, full weight-bearing was allowed. Then, after the removal of drains, a range of motion exercises and quadriceps strengthening exercises were started.

### 2.6. Statistical Analyses

The intraclass correlation coefficient was used to quantify both inter-observer and intra-observer reliabilities for radiographic assessment. A descriptive analysis was performed for all the variables, including calculation of the mean and standard deviation. Data normality was tested using a *Kolmogorov–Smirnov test*. The *paired T-test* was used to compare between the pre-operative and post-operative parameters. The *Student’s T-test* for continuous variables and chi-square tests for categorical variables were used to compare the parameters between mild varus group and severe varus group.

Statistical analyses were performed using SPSS software, version 18 (IBM Corp., Armonk, NY, USA). Statistical significance was determined as a *p*-value less than 0.05 for all analyses.

## 3. Results

A total 266 consecutive patients who underwent primary TKA surgery with the same method for degenerative osteoarthritis with varus deformity were reviewed. Among them, 80 patients were excluded who were followed up for less than 1 year. A total of 26 patients were excluded who previously underwent high tibial osteotomy or surgery for fracture. Ten patients who were diagnosed with rheumatoid arthritis were excluded. Finally, 150 patients (220 cases) were enrolled in the present study (Table 1). Among 220 cases, 111 cases are classified as mild varus group (<10°) and 109 cases are classified as severe varus group (≥10°).

Mean follow-up duration was 26.9 months. Inter-observer and intra-observer ICCs show good agreement regarding radiographic measurement reliability (>0.80). The HKA alignment is significantly decreased (*p* < 0.001). The average tibial component angle after surgery is 90.1°. A total of 21 cases (9.5%) and 3 cases (1.4%) are classified as an outlier of HKA alignment and tibial component angle, respectively. The average tibial slope before surgery is 9.3° and after surgery is 3.2° (*p* < 0.001). The average incision size is 12.3 cm and the average operative time is 75.1 min. The post-operative clinical outcomes including HSS score and WOMAC index are significantly improved compared to pre-operative values (*p* < 0.05) (Table 2).

The severe varus group shows significantly more post-operative HKA alignment outliers than the mild varus group (mild varus group—6 cases (5.4%), severe varus group—15 cases (13.8%), *p* = 0.04, Table 3). The pre-operative MPTA is significantly greater in the mild varus group (*p* < 0.001). However, the post-operative MPTA outliers did not show statistical significance (mild varus group—one case (0.9%), severe varus group—two cases (1.5%), *p* = 0.62).

## 4. Discussion

The principal finding of our study is that a favorable coronal position of the tibial component could be obtained by using preplanned proximal tibial radiographic references and intraoperative synchronizing. The severe varus group shows similar outliers of tibial component coronal position, but significantly more outliers of HKA alignment compared to the mild varus group.

Computer navigation instruments or patient-specific instruments (PSI) are helpful in post-operative limb and implant alignment [8,9]. Cheng et al. investigated the efficacy of computer-navigation-assisted surgery by meta-analysis of 41 randomized controlled trials [8]. They report that malalignment of >3° from neutral alignment in the HKA alignment occurs in fewer patients in the computer-navigation-assisted group than in the conventional group (12.2% vs. 28.3%, respectively). Schotanus et al. conduced a meta-analysis of comparison between PSI-assisted and conventional TKA [9]. They demonstrate that MRI-based PSIs show a 19% decline in outliers compared to conventional TKAs. However, these devices are not always available and have additional cost, therefore, many surgeons perform conventional jig-based TKA. Both extramedullary and intramedullary guided cut can be used, and show similar results [28,29]. However, the intramedullary system shows less accuracy in patients with tibial bowing or post-traumatic deformities [30]. The installation of extramedullary tibia cutting instruments depends on anatomical landmarks, which include intercondylar eminence, center of tibial plateau, posterior cruciate ligament, and tibial tuberosity for the proximal side, and anterior tibialis tendon, extensor hallucis longus, dorsal pedis, and intermalleolar center for the distal side [11,12,13,31]. These landmarks may not be palpable, and the surgeon’s experience is important to obtain an accurate tibial component position. We suggest that additional radiographic references (proximal tibial reference point, ML cut thickness difference) and intraoperative hip and ankle center marking by C-arm intensifier could enhance the tibial component coronal position. These additional references and center markings provide an accurate location of the extramedullary instrument and allow a second chance to check limb and component alignment after trial insertion. It is well-known that high-grade pre-operative varus deformity is associated with residual post-operative varus alignment [32]. In the current study, the severe varus group shows more HKA alignment outliers than the mild varus group, which is concurrent with previous studies. However, MPTA outliers are not significantly different between both groups. Previous studies suggest that the increasing severity of pre-operative varus deformity is associated with complex bone cuts to restore a neutral mechanical alignment [1,33]. According to our findings, we think our methods aid in obtaining tibial component neutral alignment, regardless of the severity of pre-operative varus deformity.

Proximal tibial reference points of all cases exist between the center of intercondylar eminence and the lateral tibial spine in the current study. Kim et al. [34] suggest that lateral intercondylar spine rather than the center of intercondylar eminence should be used as a reference for the proximal tibial side in extramedullary guided tibial cut, in order to obtain a neutral component position. We agreed that routine use of the center of intercondylar eminence as a proximal reference point has a chance to highlight the varus position of the tibial component. However, we think an individualized proximal tibial reference point is better than routine use of lateral intercondylar spine. A further comparative study is needed to find the most reliable reference point of the proximal tibia.

This study has several limitations. First, it was not a comparative study with other methods. Second, the follow-up period was relatively short, and further studies are needed to evaluate implant survival. Third, there is not enough discussion on femur cutting with emphasis on increasing the accuracy of tibia cutting. Forth, we only focused on correcting coronal malalignment, and we need to discuss the correction technique for the sagittal plane as well. Fifth, in addition to the bone cutting, the collateral ligament and soft tissue were involved in the gap-balancing, but there was not sufficient information regarding this.

## 5. Conclusions

The measurement of proximal tibial radiographic references and checking C-arm-guided intraoperative hip and ankle centers could be helpful in obtaining a favorable coronal position of the tibial component for the extramedullary guided tibial cut in TKA.

## Figures and Tables

**Table 1:** Demographic and preoperative radiographic parameters in 220 cases.

Age, year ^a^	70.55 ± 7.21
Sex, male:female	46:174
Direction, right:left	107:113
Proximal tibial reference point	
Center	157 (71.4%)
In-between	24 (10.9%)
Lateral tibial spine	39 (17.7%)

^a^ Values are presented as mean ± standard deviation (range).

**Table 2:** Comparison of clinical and radiographic data between pre- and post-operation.

	Pre-Operation	Post-Operation	*p* Value
HKA alignment, °	10.4 ± 6.4	1.9 ± 1.7	<0.001
MPTA, °	83.9 ± 3.3	90.1 ± 0.9	<0.001
Tibial slope, °	9.3 ± 4.4	3.2 ± 2.1	<0.001
HSS score	38.7 ± 11.9	77.7 ± 9.9	<0.001
WOMAC index	68.0 ± 7.1	28.2 ± 5.4	<0.001

HKA: hip–knee–ankle, MPTA: medial proximal tibial angle, HSS: Hospital for Special Surgery, WOMAC: Western Ontario Mac-Master University.

**Table 3:** Comparison of measurements between mild varus group and severe varus group.

	Mild Varus Group (<10°)	Severe Varus Group (≥10°)	*p* Value
Number of cases	111	109	
Age	70.2 ± 7.0	70.8 ± 7.4	0.517
Direction (R:L)	57:54	50:59	0.422
Pre-operative HKA alignment (°)	5.7 ± 2.5	15.0 ± 5.8	**<0.001**
Post-operative HKA alignment (°)	1.4 ± 1.7	2.4 ± 2.1	**<0.001**
HKA alignment outliers, n (%)	6 (5.4%)	15 (13.8%)	**0.04**
Pre-operative MPTA (°)	85.1 ± 2.9	82.7 ± 3.1	**<0.001**
Post-operative MPTA (°)	90.2 ± 0.8	90.1 ± 1.0	0.654
MPTA outliers, n (%)	1 (0.9%)	2 (1.5%)	0.62
Pre-operative tibial slope (°)	8.8 ± 4.5	9.9 ± 4.3	0.065
Post-operative tibial slope (°)	3.1 ± 2.0	3.4 ± 2.3	0.394

HKA: hip–knee–ankle, MPTA: medial proximal tibial angle.

## Data Availability

Not applicable.
